# Plasmonic Modulators in Cryogenic Environment Featuring Bandwidths in Excess of 100 GHz and Reduced Plasmonic Losses

**DOI:** 10.1021/acsphotonics.4c00507

**Published:** 2024-06-28

**Authors:** Dominik Bisang, Yannik Horst, Maurus Thürig, Kiran Menachery, Stefan M. Koepfli, Manuel Kohli, Eva De Leo, Marcel Destraz, Valentino Tedaldi, Nino Del Medico, Claudia Hoessbacher, Benedikt Baeuerle, Wolfgang Heni, Juerg Leuthold

**Affiliations:** † Institute of Electromagnetic Fields, ETH Zurich, 8092 Zurich, Switzerland; ‡ Polariton Technologies AG, 8134 Adliswil, Switzerland

**Keywords:** silicon photonics, modulator, plasmonics, high speed, cryogenic

## Abstract

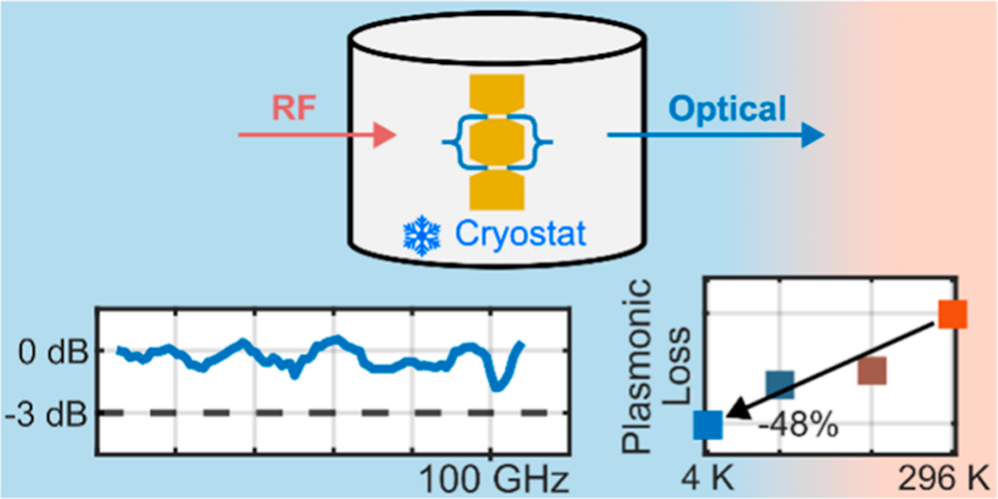

Cryogenic quantum applications have a demand for an ever-higher number of interconnects and bandwidth. Photonic links are foreseen to offer data transfer with high bandwidth, low heat load, and low noise to enable the next-generation scalable quantum computing systems. However, they require high-speed and energy-efficient modulators operating at cryogenic temperatures for electro-optic signal conversion. Here, plasmonic organic electro-optic modulators operating at 4 K are demonstrated with a >100 GHz bandwidth, drive voltages as low as 96 mV, and a significant reduction in plasmonic propagation losses by over 40% compared to room temperature. Up to 160 Gbit/s and 256 Gbit/s cryogenic electro-optic signal conversion are demonstrated by performing data experiments using a plasmonic Mach–Zehnder modulator at around 1528 nm and a plasmonic ring-resonator modulator at around 1285 nm, respectively. This work shows that plasmonic modulators are ideally suited for future high-speed, scalable, and energy-efficient photonic interconnects in cryogenic environments.

## Introduction

**Figure 1:**
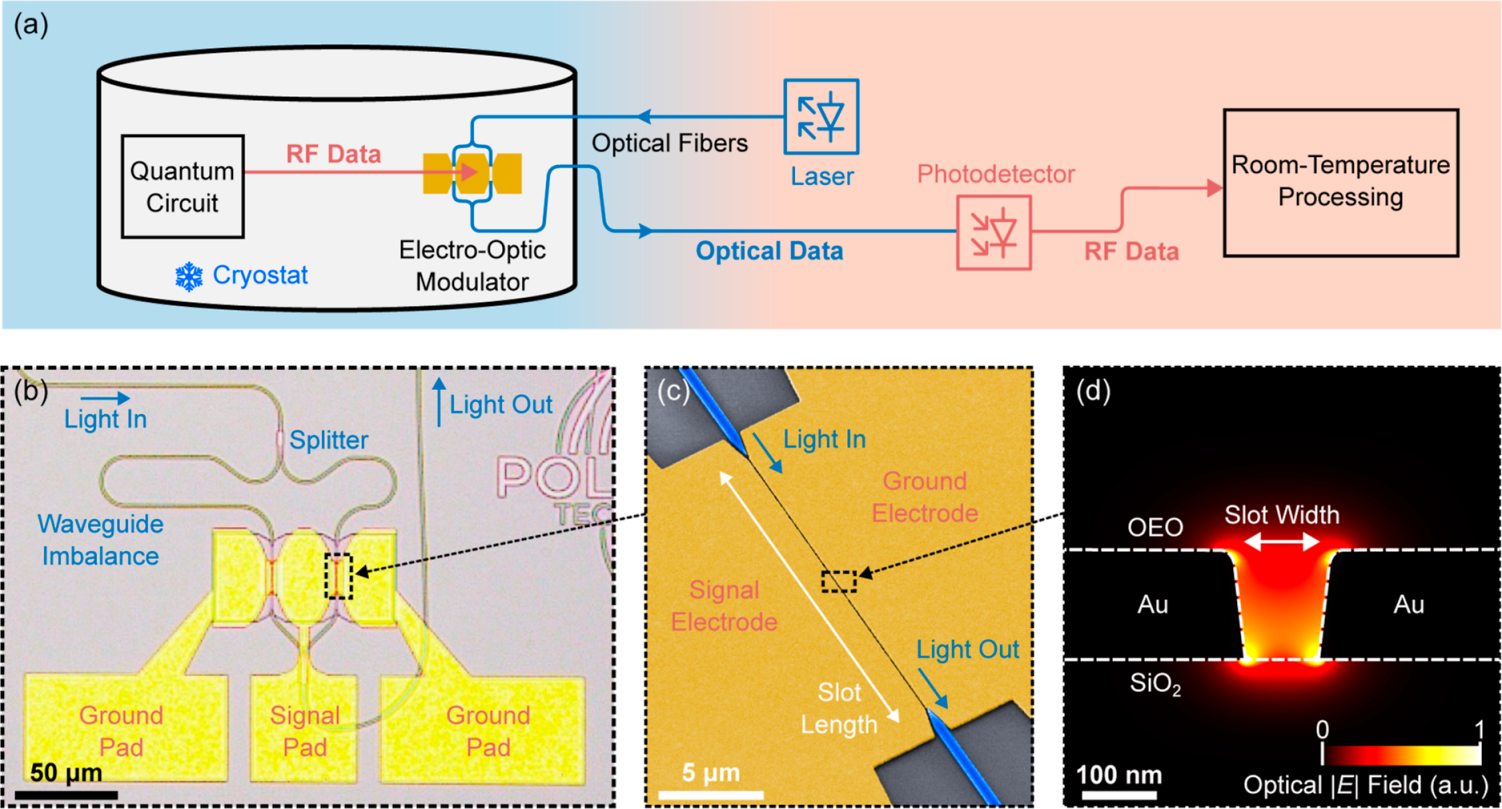
(a) Schematic visualization of an envisioned high-speed optical link connecting a cryogenic quantum circuit with room-temperature processing. (b) Microscope image of a plasmonic MZM. Both arms of the MZM contain a plasmonic phase shifter. (c) False-color scanning electron microscope image of a typical plasmonic phase shifter with silicon waveguides as light input and output. (d) Schematic visualization of a plasmonic slot cross section with the optical field distribution inside the slot. The plasmonic slot is formed by Au on top of SiO_2_ and filled with a nonlinear OEO material.

Cryogenic electro-optic modulators are essential building blocks for the next-generation scalable quantum computing systems. They are needed in optical interconnects to transfer data between cryogenic quantum devices and room-temperature counterparts. The growing importance and complexity of quantum computing^1,2^ or single flux quantum (SFQ) logic^3^ requires high-speed, energy-efficient, and scalable solutions to interconnect the cryogenic environment. Yet, the current approach of utilizing rigid radio frequency electrical cables presents limitations in upscaling due to heat load, cost, and complexity of electrical wiring.^4^ Optical fibers have been proposed as an alternative due to numerous advantages^5−7^ such as low-loss over kilometer distances and a vast optical bandwidth, enabling transmission of multiple channels via a single fiber using wavelength multiplexing. In comparison to RF coaxial cables, optical fibers have massively lower heat load, reduced noise, and lower production costs. The key challenges to employ optical fiber interconnects to cryogenic temperatures are cryo-compatible electro-optical converters, i.e., modulators. Ideally, such a modulator should not only offer operation at cryogenic temperature but also operate with low energy consumption per bit, offer a small footprint, and have the largest possible bandwidth. An envisioned optical link from a cryostat to room temperature is sketched in Figure 1a, where electrical signals generated from a quantum or SFQ logic circuit are converted to optical signals using an electro-optic modulator inside the cryogenic environment.

Several electro-optic modulators operating at cryogenic temperatures have been demonstrated, for example, silicon microdisk modulators,^8^ silicon Mach–Zehnder modulators (MZMs) with PIN junctions modulated by the DC Kerr effect,^9^ graphene-based ring-resonator modulators (RRMs) on silicon nitride,^10^ barium titanate RRM on silicon and silicon nitride,^11^ silicon RRM on a CMOS platform,^12^ commercially available lithium niobate phase modulators,^6^ quantum wells in InP-on-Si RRM,^13^ or lithium niobate MZM with superconducting electrodes.^7^ Recently, cryogenic modulators exploiting the linear electro-optic coefficient in organic electro-optic (OEO) materials have been introduced.^14,15^ Operation of photonic silicon–organic hybrid (SOH) and plasmonic organic hybrid modulators at 4 K with data rates of 50 GBd and 128 GBd 2-level pulse amplitude modulation (2PAM) has been shown. In a follow-up publication, a 1 mm-long SOH MZM achieved 70 GBd 4PAM data transmission.^16^ These results are in line with earlier findings that the linear electro-optic effect in OEO materials is an efficient alternative for cryogenic applications as it maintains its high nonlinearities down to cryogenic temperatures.^17^ While all of these demonstrations are impressive and give testimony of rapid progress, the question remains as to what the full potential might be. Particularly, the plasmonic solution with devices of only a few micrometers in length seems attractive. They have already shown excellent characteristics at room temperature with electro-optic bandwidths exceeding 500 GHz,^18^ symbol rates in data transmission of 256 GBd using an IQ modulator,^19^ and energy-efficient 120 Gbit/s transmission with a peak-to-peak driving voltage of 178 mV.^20^ While traditionally, the advantage of compactness comes at the cost of higher optical loss, a low on-chip insertion loss of 1.5 dB was recently demonstrated using a plasmonic RRM.^21^

In addition, plasmonics holds promise to benefit from reduced optical losses at cryogenic temperatures. Previous demonstrations show a reduction of plasmonic losses at cryogenic temperatures using silver at visible^22−24^ and near-infrared wavelengths.^25^ Gold, as commonly used in plasmonic modulators, was demonstrated to have lower plasmonic losses at cryogenic temperatures at visible wavelengths,^26,27^ and indeed, these results are promising for a cryogenic plasmonic modulator. Yet, studies have also found that the reduction of plasmonic losses strongly depends on the metal crystallinity and roughness.^22,26^ The question then is, if the plasmonic platform can provide modulators that feature low optical losses and highest-speed operation with the smallest driving voltages.

In this work, we demonstrate a plasmonic MZM and a plasmonic RRM operated at cryogenic temperatures. We show that plasmonic modulators are ideally suited for cryogenic operation since the propagation loss is reduced by more than 40% compared to room temperature, and they feature an electro-optic 3 dB bandwidth exceeding 100 GHz. The half-wave voltage increases by only 11% compared to room temperature. We demonstrate up to 80 GBd 4PAM (160 Gbit/s) and 128 GBd 4PAM (256 Gbit/s) cryogenic electro-optic signal conversion by performing data experiments using a plasmonic MZM at around 1528 nm and, for a first time, a plasmonic RRM in the O band at around 1285 nm, respectively. Furthermore, we present with both devices 16 Gbit/s with 96 and 191 mV peak-to-peak drive voltage for energy-efficient data transmission out of the cryogenic environment. This demonstrates how plasmonic modulators offer a promising low-voltage, high-speed solution for electro-optic signal conversion at cryogenic temperatures.

This paper is an extension of the work previously published at the ECOC 2022, Basel, Switzerland.^15^

## Device Concept

The plasmonic modulators for cryogenic applications presented in this work are based on plasmonic phase shifters, as shown in Figure 1c. A photonic TE mode in a silicon waveguide is coupled to a plasmonic mode in a horizontal plasmonic metal–insulator–metal (MIM) slot configuration^28^ using waveguide tapering. A schematic cross section of the slot with the simulated optical field is shown in Figure 1d. The slot is formed by gold, which simultaneously confines the light into the slot and serves as electrodes for the electrical signal. Electro-optic modulation is achieved by using the linear electro-optic effect (*r*_33_) of an OEO material inside the slot. More specifically, the material HLD was used,^29^ with an in-device *r*_33_ of 159 pm/V measured at room temperature.

Two plasmonic modulators are presented in this work. The first is an imbalanced plasmonic MZM as shown in the microscope image in Figure 1b. The plasmonic MZM is designed for push–pull operation. In both arms of the MZM is a plasmonic phase shifter consisting of a 105 nm-wide and 15 μm-long plasmonic slot. Silicon photonic waveguides of different lengths are used to feed light into the plasmonic phase shifters. This imbalance between the two interferometer arms allows adjustment of the modulator’s operation point by changing the wavelength of a tunable laser source (TLS). This configuration was specifically chosen for cryogenic operation. With a balanced MZM, additional electrical tuning would be required by, e.g., thermo-optic phase shifters, which typically consume power in the order of 10 mW for a phase shift of π at room temperature. Therefore, using an imbalanced MZM has the advantage that the thermal load to the cryostat can be reduced. Light is coupled from an optical fiber into the waveguides by using grating couplers.

The second is a plasmonic RRM^21^ as shown in a microscope image later in this paper. It consists of a silicon ring cavity coupled to a silicon waveguide via a directional coupler. Within the silicon ring, a plasmonic phase shifter is placed to change the ring resonance frequency by electrically introducing a phase shift, thereby modulating an optical signal. The ring modulator is partially covered with an oxide cladding and partially opened in the region of the plasmonic phase shifter. This is to allow for electrical contact and for the implementation of additional photonic phase shifters (e.g., a thermo-optic phase shifter) to adjust the operating point of the RRM. Yet, for the device intended for cryogenic operation, no thermo-optic phase shifter was implemented to avoid any additional thermal load to the system. The plasmonic RRM has a plasmonic slot length of 10 μm, a plasmonic slot width of 105 nm, and a photonic ring length of 123 μm. The overall device footprint is 30 μm × 40 μm without contacting electrodes and fiber-to-chip couplers, making it a promising candidate for dense multichannel integration.^21^

## Results and Discussion

### Plasmonic Propagation Losses at Cryogenic Temperatures

This section reports a 40–50% reduction of plasmonic propagation losses at cryogenic temperatures compared to room-temperature measurements.

The plasmonic losses were measured in plasmonic phase shifters based on a horizontal plasmonic MIM slot configuration, as shown in Figure 1d. Devices with varying lengths and widths of plasmonic slots were placed within a cryogenic probe station. A microscope image of exemplary plasmonic slot devices and a schematic drawing of the experimental setup used to measure the plasmonic propagation loss in the cryostat are shown in the Supporting Information. Light is coupled in and out of the silicon photonics waveguides by positioning a fiber array over grating couplers. The cryogenic probe station has a base temperature of 3.2 K, which is measured via a sensor mounted on the sample stage close to the chip. We estimate a temperature difference from the sample stage to the chip in the order of one Kelvin, thereby having the chip at roughly 4 K when the probe station is at base temperature. The chip temperature can further be changed between base temperature and room temperature by heating the sample stage with resistive heaters.

**Figure 2:**
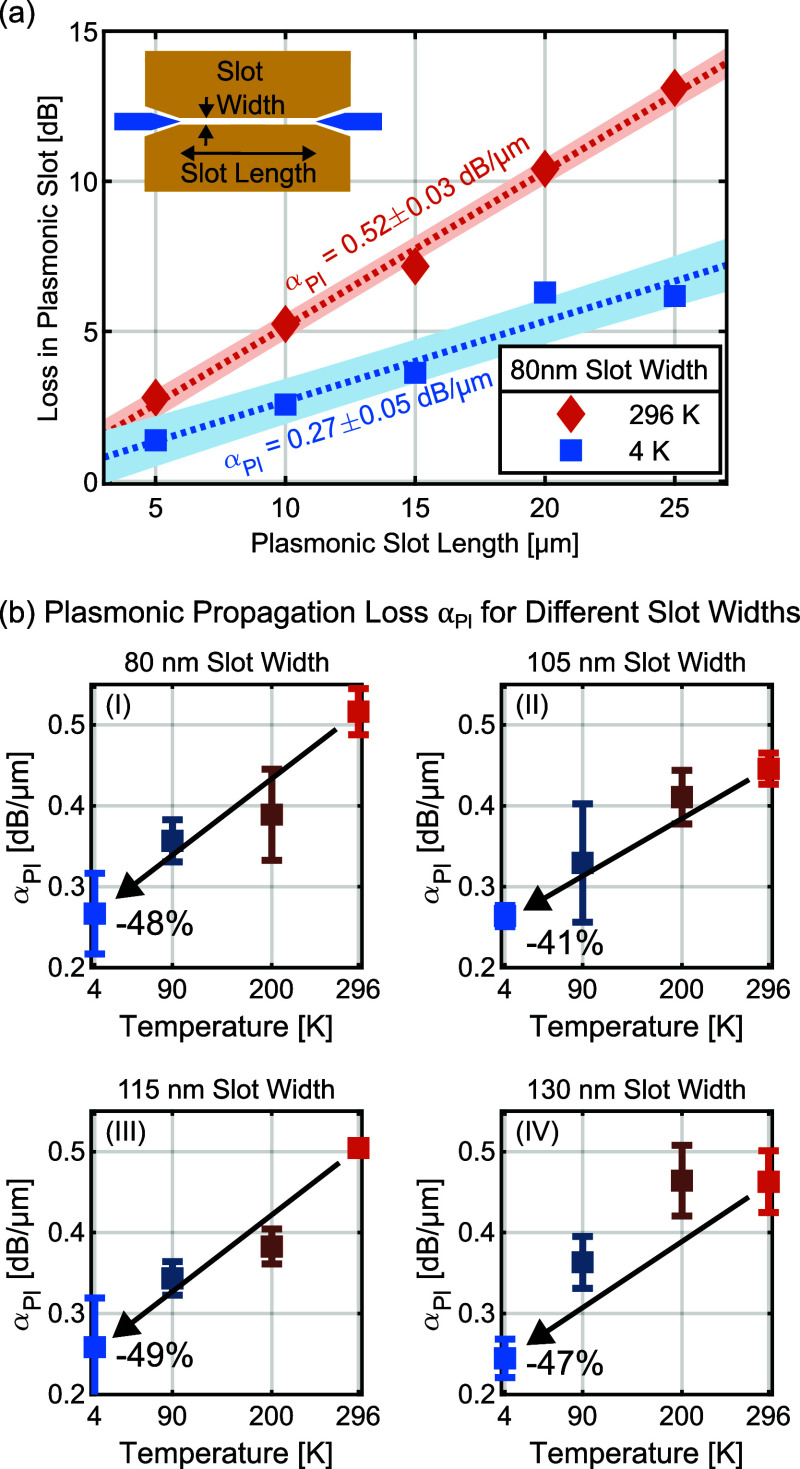
(a) Length-dependent plasmonic loss of five devices with different plasmonic slot lengths at 4 K (blue squares) and 296 K (red diamonds). The dashed lines represent linear fits through the measured data. The areas surrounding the dashed lines are one-sigma confidence intervals of the fits. The plasmonic propagation loss α_Pl_ with one-sigma uncertainty (text next to dashed line) is extracted from the slope of the linear fit. (b) Plasmonic propagation loss α_Pl_ with one-sigma uncertainty error bars measured at 4, 90, 200, and 296 K for plasmonic slots with widths of (I) 80 nm, (II) 105 nm, (III) 115 nm, and (IV) 130 nm. A reduction of the plasmonic propagation loss of over 40% was found for all four slot widths when cooling down the devices from 296 to 4 K.

The plasmonic propagation loss was extracted by measuring the passive optical transmission of five individual devices with 5, 10, 15, 20, and 25 μm length of the plasmonic slot. By performing a linear fit of the plasmonic loss versus the plasmonic slot length, the propagation losses can be extracted. This procedure is indicated in Figure 2a for a group of five devices with 80 nm-wide plasmonic slots at 4 and 296 K in the C band. It is found that the plasmonic propagation losses decrease from 0.52 dB/μm at 296 K (room temperature) to 0.27 dB/μm at 4 K. The one-sigma uncertainties are also shown in Figure 2a. The uncertainty estimation is described in the Supporting Information.

This methodology was further used to measure the plasmonic propagation loss for plasmonic slots of varying widths, namely, 80, 105, 115, and 130 nm, at temperatures of 4, 90, 200, and 296 K. The measured optical losses for each individual device are shown in the Supporting Information. The resulting plasmonic propagation losses α_Pl_ are presented in Figure 2b, where each data point corresponds to a linear fit with five devices of different plasmonic slot lengths, corresponding to a total of 20 devices measured at four temperatures. Clearly, the plasmonic propagation loss in the MIM slots decreases by 40–50% when cooling the devices from room temperature to 4 K. From the plots, one can observe a slight trend toward lower losses with increasing slot width. This would be in line with our previous findings at room temperature.^30,31^

### Cryogenic Characterization of the Plasmonic MZM

In this section, we report that the electro-optic bandwidth of the OEO material does not decrease at cryogenic temperatures and remains flat for frequencies beyond 100 GHz. Furthermore, we find that the half-wave voltage *V*_π,50 Ω_ increases from 3.4 to 3.7 V for the plasmonic MZM when going from room temperature to cryogenic temperatures of 4 K, with a maximum of 4.0 V at around 50 K (a 20% increase).

The results were obtained with a plasmonic MZM, as shown in the microscope image in Figure 1b. In this device, light is coupled from an optical fiber into the waveguides by using grating couplers. To reduce the impact of mechanical vibrations inside the cryostat on the optical coupling, the fiber array was glued on the chip with cryo-compatible epoxy.

**Figure 3:**
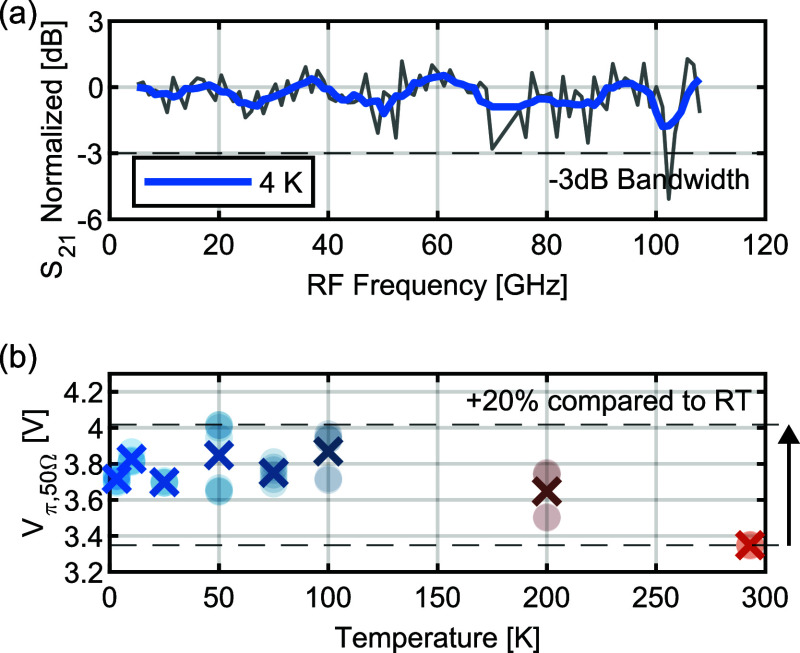
(a) Normalized electro-optic frequency response of the plasmonic MZM at 4 K, measured (thin gray line) and moving-average-filtered (thick blue line). (b) Half-wave voltage matched to a 50 Ω signal source *V*_π,50Ω_ of the plasmonic MZM at different temperatures, measured (pale dots) and averaged over all measurements at one temperature (crosses). The dashed lines indicate the room-temperature (RT) value and 1.2 times the RT value as references for comparison.

To characterize the active performance of the plasmonic MZM, the electro-optic bandwidth of the modulator at 4 K is investigated. The response is measured by applying an RF tone at a single frequency in the GHz range and an optical carrier at 1532.5 nm. Then, the peak-to-sideband ratio is measured in an optical spectrum analyzer (OSA). The full setup is described in the Supporting Information. The normalized peak-to-sideband ratio versus RF frequency at 4 K is shown in Figure 3a as a thin gray line. The thick blue line shows a five-point moving average over the measurement data for better visibility in the presence of oscillations due to noise and missing RF probe calibration above 67 GHz. The electro-optic 3 dB bandwidth of the plasmonic modulator at 4 K is larger than 100 GHz, indicating no bandwidth penalty compared to room-temperature measurements.

Next, we measured the half-wave voltage *V*_π_ at different temperatures. For this purpose, an overmodulation setup was used with a 100 kHz electrical signal (see Supporting Information). In Figure 3b, the half-wave voltage matched to a 50 Ω signal source *V*_π,50Ω_ at different temperatures is shown. The pale dots show the measured values, and the crosses show an average over all measured values at one temperature. Note that the plasmonic modulators are designed as high-impedance loads, efficiently utilizing twice the voltage provided by a 50 Ω signal source.^32^ Therefore, the measured half-wave voltage by a DC or high-impedance source corresponds to 2 times *V*_π,50Ω_. At room temperature, we find a *V*_π,50Ω_ of 3.35 V. When cooling the device down toward 4 K, only a small degradation of less than +20% compared to room temperature was observed, as indicated by the dashed line. The maximum observed *V*_π,50Ω_ was 4.02 V at 50 K; below this temperature, it decreased to an average of 3.72 V at 4 K. This temperature-induced change could in principle originate from temperature-dependent material properties of the OEO, although Schwarzenberger et al.^16^ report an identical *V*_π_ at both room temperature and at 11 K using an OEO modulator.

### Cryogenic Data Transmission Experiments with Plasmonic MZM

This section demonstrates data transmission experiments of up to 160 Gbit/s by using a plasmonic MZM operated at 4 K at around 1528 nm wavelength. Furthermore, operations with reduced electrical drive voltages down to 96 mV_PP_ are shown to mimic the low signal levels typically available for quantum applications and necessary to achieve reduced thermal dissipation. This enables cryogenic optical data transmission with low on-chip electrical energy consumption per bit.

**Figure 4:**
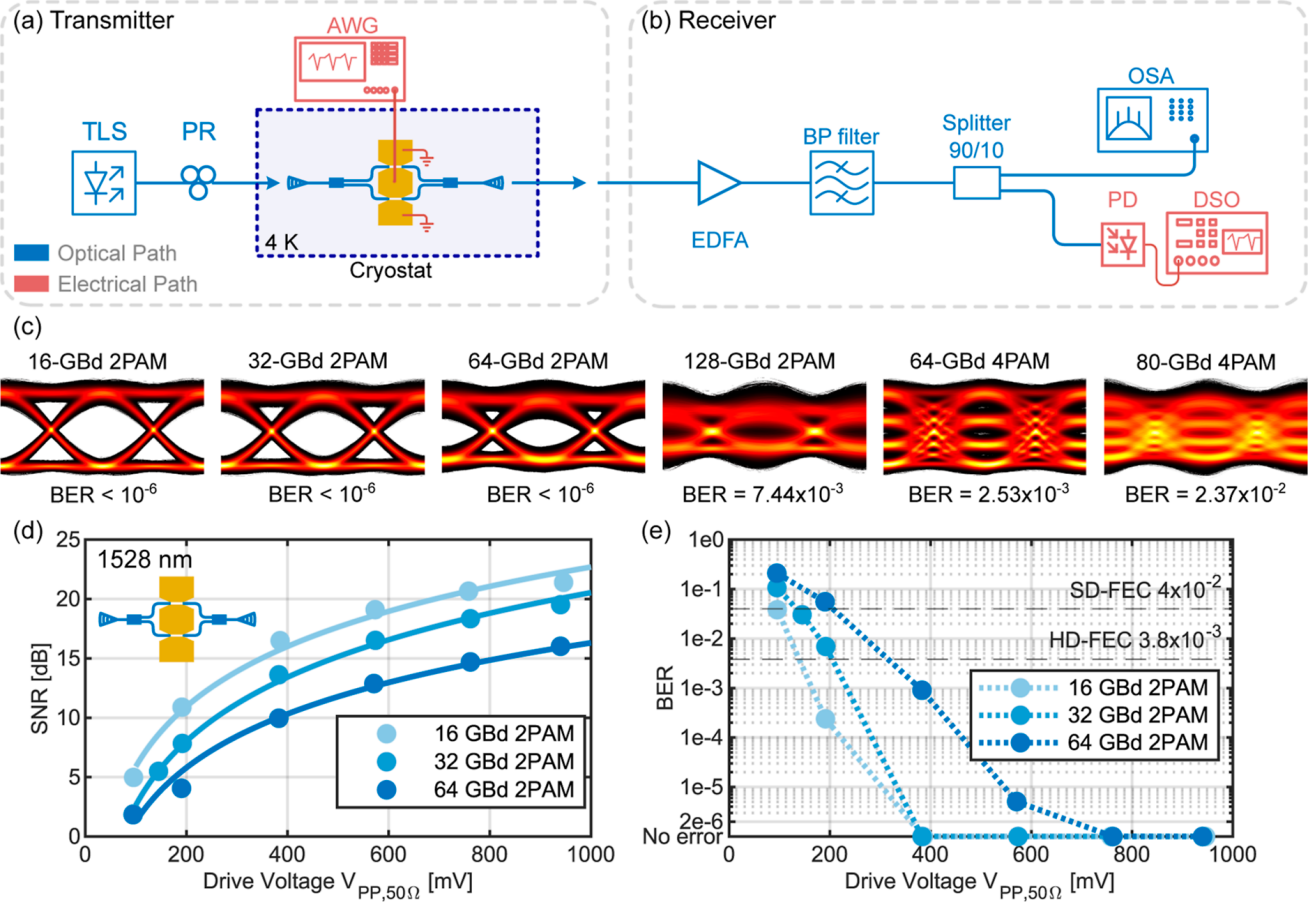
Data transmission experiments with the plasmonic MZM operated at 4 K and around 1528 nm optical carrier wavelength. (a,b) Schematic drawing of the experimental setup used for data transmission consisting of the (a) transmitter and (b) receiver. (c) Recorded eye diagrams with BER for 10^6^ transmitted 2PAM and 4PAM symbols using the plasmonic MZM. (d) Measured signal-to-noise (SNR) of transmitted 16, 32, and 64 GBd 2PAM signals as a function of the applied effective peak-to-peak drive voltage *V*_PP,50Ω_. The solid lines are quadratic fits through the measured SNRs. (e) Measured BER of the transmitted 5 × 10^5^ symbols after applying digital signal processing (DSP). The hard-decision forward error correction (HD-FEC) and soft-decision forward error correction (SD-FEC) limits are shown by dashed gray lines. The dotted line is a guide to the eye. TLS, tunable laser source; PR, polarization rotator; AWG, arbitrary waveform generator; EDFA, erbium-doped fiber amplifier; BP filter, band-pass filter; OSA, optical spectrum analyzer; PD, photodiode; DSO, digital sampling oscilloscope.

The setup used for the data experiments is schematically shown in Figure 4a,b. On the transmitter side as shown in Figure 4a, pseudorandom electrical 2PAM and 4PAM data were generated outside the cryostat with a 256 GSa/s arbitrary waveform generator (AWG) with 70 GHz bandwidth. The electrical peak-to-peak driving voltage matched to a 50 Ω load *V*_PP,50Ω_ was 1.0 V. The electrical signal is fed into the cryostat to the plasmonic modulator through a 67 GHz RF feedthrough, introducing additional RF losses. No RF amplifier was used in the electrical chain. The optical carrier was generated using a TLS at a wavelength around 1528 nm with 10 dBm output power. A polarization rotator was used to optimize the polarization for maximum transmission through the device. The modulator was operated inside the cryostat at 4 K as described in the previous section with a fiber array glued onto the grating couplers. The fiber-to-fiber insertion losses were around −34 dB. The high losses are mainly attributed to a poor fiber-to-chip coupling inside of the cryostat. The on-chip device losses are estimated to be −5.9 dB. On the receiver side, see Figure 4b, the modulated optical signal is then amplified using an erbium-doped fiber amplifier and optically band-pass-filtered (BP filter). The signal is then split up using a 90/10 splitter where 90% of the light is fed into a 145 GHz photodiode (PD) for the optoelectrical conversion, and the electrical signal is then sampled and stored in a digital sampling oscilloscope for later offline DSP. The other 10% of the light is fed into an OSA for monitoring. The offline DSP consists of a matched filter, timing recovery, and a static T/2-spaced feed-forward equalizer trained by a data-aided least mean square algorithm with 99 filter taps for the MZM and 151 filter taps for the RRM.

Recorded eye diagrams using the plasmonic MZM at 4 K are shown for symbol rates of 16 to 128 GBd 2PAM (16 to 128 Gbit/s), 64 GBd 4PAM (128 Gbit/s), and 80 GBd 4PAM (160 Gbit/s), see Figure 4c. For these eye diagrams, 10^6^ symbols are transmitted. Error-free data transmission was found up to 64 GBd 2PAM.

Furthermore, the influence of reduced drive voltage on the data transmission was investigated. Figure 4d shows the SNR and Figure 4e shows the bit-error ratio (BER). Both are derived from the measured and processed data and plotted as a function of the effective electrical peak-to-peak drive voltage *V*_PP,50Ω_. The effective drive voltage with subtracted RF cable losses was calibrated without RF feedthrough into the cryostat and RF probe.^32^ For a peak-to-peak drive voltage of 96 mV, 16 GBd 2PAM had a BER below the SD-FEC limit of 4 × 10^–2^. For 385 mV peak-to-peak drive voltage, 16 GBd and 32 GBd 2PAM remained error-free with 5 × 10^5^ transmitted symbols, and 64 GBd achieved a BER below the HD-FEC limit of 3.8 × 10^–3^.

### Cryogenic Operation of the Plasmonic RRM

**Figure 5:**
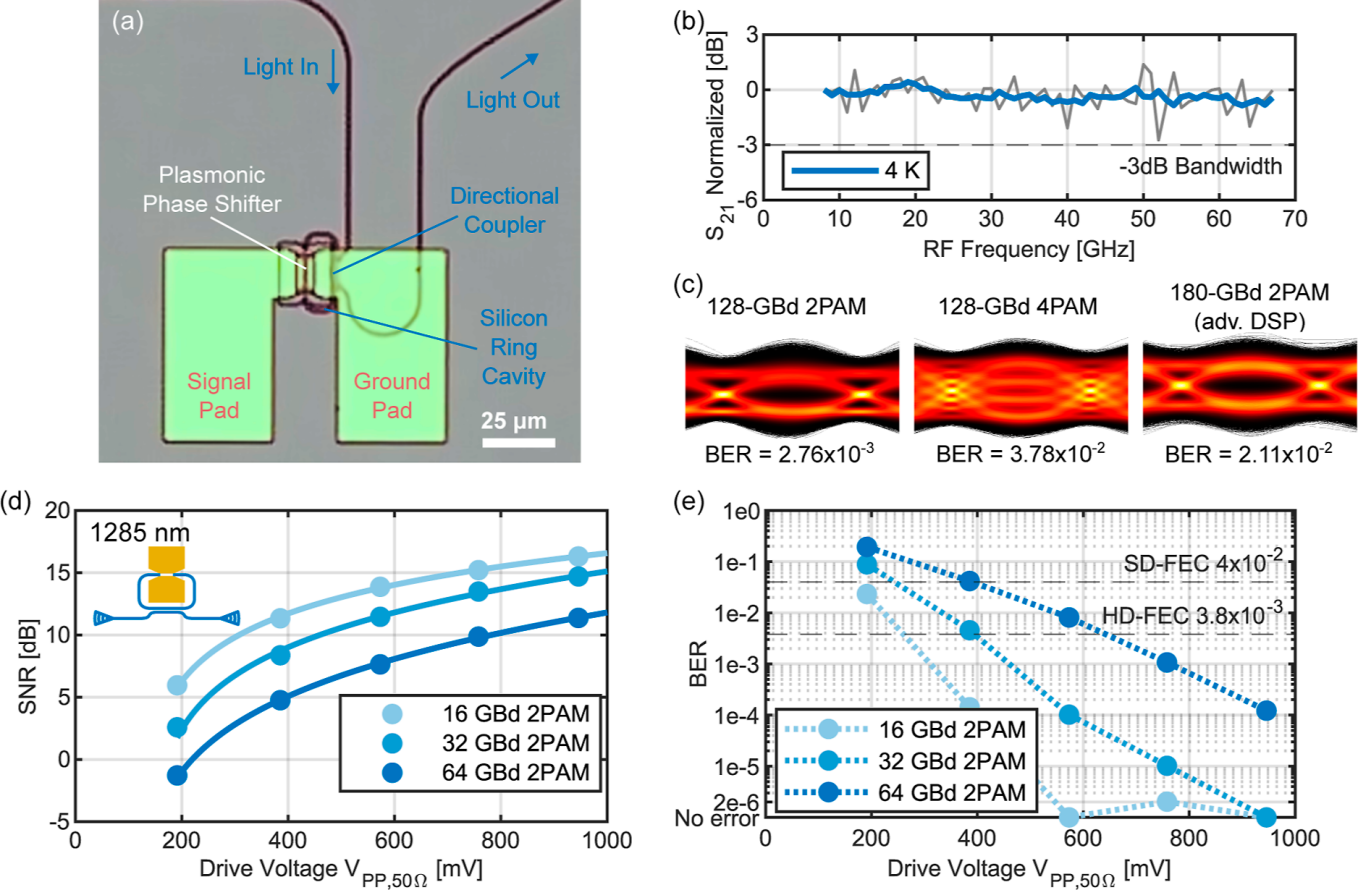
Plasmonic RRM operated at 4 K and around 1285 nm optical carrier wavelength. (a) Microscope image of a plasmonic RRM. (b) Normalized electro-optic frequency response of the plasmonic RRM at 4 K, measured (thin gray line) and moving-average-filtered (solid line). (c) Recorded eye diagrams with BER for 4 × 10^6^ transmitted 2PAM and 4PAM symbols using the plasmonic RRM. For 180 GBd 2PAM, more advanced DSP was used. (d) Measured SNR of transmitted 16, 32, and 64 GBd 2PAM signals as a function of the applied effective peak-to-peak drive voltage *V*_PP,50Ω_ of the plasmonic RRM. The solid lines are quadratic fits through the measured SNRs. (e) Measured BER of the transmitted 5 × 10^5^ symbols after applying DSP. The HD-FEC and SD-FEC limits are shown by dashed gray lines. The dotted line is a guide to the eye.

A plasmonic RRM,^21^ as shown in the microscope image in Figure 5a, was used to demonstrate cryogenic high-speed modulation in the O band at 1285 nm. The spectral tuning per volt is 110 pm/V at room temperature and 31 pm/V at 4 K, measured by a shift in the transmission spectrum due to an applied voltage. This corresponds to half-wave voltages *V*_π,50Ω_ of 7.9 V at room temperature and 27.1 V at 4 K. Note that the device suffered from contacting issues at cryogenic temperature, and the degradation of *V*_π,50Ω_ might be related to a damaged electrical contact. Another plasmonic RRM measured in the C band showed a 27% increase of *V*_π,50Ω_ when cooling down to 4 K, reproducing the MZM results discussed earlier. The fiber-to-fiber loss of the chip was approximately −22 dB. The electro-optic frequency response of the plasmonic RRM at 4 K was measured for up to 67 GHz and remained flat as shown in Figure 5b.

The experimental setup used for the data transmission is mostly identical to the one shown in Figure 4a,b, except using the O band RRM inside the sketched cryostat in Figure 4a and replacing the instruments by components operating in the O band instead of the C band. The optical carrier was generated at around 1285 nm with 4.3 dBm power, and the effective electrical peak-to-peak drive voltage *V*_PP,50Ω_ was 1.0 V. In Figure 5c, recorded eye diagrams for 4 × 10^6^ transmitted symbols using the plasmonic RRM at 4 K are shown. We demonstrate symbol rates of 128 GBd 2PAM (128 Gbit/s) with a BER below the HD-FEC limit and 128 GBd 4PAM (256 Gbit/s) and 180 GBd 2PAM (180 Gbit/s), both below the SD-FEC limit. For 180 GBd 2PAM, more advanced offline DSP was used with an additional nonlinear equalization based on a 7-symbol pattern mapping.^33^ The limited bandwidth of the AWG of around 70 GHz leads to reduced signal quality at high symbol rates.

Furthermore, the influence of reduced drive voltage on the data transmission was also investigated for the plasmonic RRM. The optical carrier power was here reduced to 0 dBm. Figure 5d shows the SNR as a function of *V*_PP,50Ω_ for 16 GBd, 32 GBd, and 64 GBd 2PAM data transmission, and Figure 5e shows the corresponding BER. As can be seen, for 16 GBd and a *V*_PP,50Ω_ of 191 mV, a BER below the SD-FEC limit is possible. For a *V*_PP,50Ω_ of 573 mV, the transmission of 5 × 10^5^ symbols with 16 GBd remained error-free, with 32 GBd, the received signal stayed below the HD-FEC limit, and with 64 GBd, it was still below the SD-FEC limit. Notably, there is a difference of approximately 5 dB SNR and therefore higher BER for the same driving voltage, between the plasmonic MZM around the C band at 1528 nm and the plasmonic RRM in the O band at 1285 nm. This is attributed to better performing C-band equipment when compared to the O-band equipment, namely, the TLS, optical amplifier, and PD.

### Electrical Energy Consumption per Bit and Literature Comparison

Finally, we estimate the energy efficiency of the plasmonic modulators at cryogenic temperatures and compare the presented results with other electro-optic modulators found in the literature. Following the procedure described by Heni et al.,^32^ the energy consumption per bit *E*_bit_ can be calculated by  for 2PAM and  for 4PAM,^34^ where *C*_dev_ is the capacitance of the modulator. It must be noted that a plasmonic modulator is not a 50 Ω-terminated but a high-impedance device, yielding a doubling of *V*_PP_ on the device when using a standard 50 Ω source, i.e., *V*_PP_ = 2*V*_PP,50Ω_. Having an estimated device capacitance of 25 fF of the plasmonic MZM including RF pads, the electrical energy consumption for 16 GBd 2PAM with a *V*_PP,50Ω_ of 96 mV is as low as 230 aJ/bit, for 128 GBd 2PAM with a *V*_PP,50Ω_ of 1.0 V, the *E*_bit_ is 25 fJ/bit, and for 80 GBd 4PAM with a *V*_PP,50Ω_ of 897 mV, the *E*_bit_ is 5.6 fJ/bit. For the plasmonic RRM in the O band, we assume a 13 fF device capacitance, which is about half of the plasmonic MZM device capacitance as the plasmonic RRM has only one plasmonic phase shifter. Using this estimation with a *V*_PP,50Ω_ of 1.0 V, the *E*_bit_ is 13 fJ/bit for 128 GBd and 180 GBd 2PAM and 3.6 fJ/bit for 128 GBd 4PAM. An estimation of the overall active heat load imposed by the plasmonic modulators, which further involves the optical power, can be found in the Supporting Information.

In Table 1, the results of this work are compared to other electro-optic cryogenic modulators. This work presents, to the best of our knowledge, the first cryogenic modulator with an electro-optic bandwidth >100 GHz and with 160 Gbit/s (80 GBd 4PAM) the highest reported line rate at cryogenic temperatures around the C band. Furthermore, with both 230 aJ/bit at 16 Gbit/s 2PAM and 5.6 fJ/bit at 80 GBd 4PAM, we show improvements in terms of energy efficiency compared to experiments with similar line rates. Also, we show the highest line rate at cryogenic temperatures in the O band with 256 Gbit/s (128 GBd 4PAM).

**Table 1:** Overview of Cryogenic Electro-Optic Modulators Found in the Literature

device and materials	bandwidth (GHz)	data line rate (Gbit/s)	modulation voltage (*V*_PP_)	electrical energy	modulator length^b^ (μm)	optical wavelength
silicon microdisk on SOI^8^	>20	10	1.8		11	1538 nm
MZM with silicon PIN junctions modulated by DC Kerr effect on SOI^9^	>1.5				4′500	1548 nm
graphene-based RRM on Si_3_N_4_^10^	14.7	20	3		251	1586 nm
BaTiO_3_ RRM, Si waveguides^11^	30	20	1.7	45 fJ/bit	154	1550 nm
silicon spoked-ring modulator, 45 nm CMOS platform^12^	2.75	20	1.5		31	1293 nm
LiNbO_3_ waveguide phase modulator^6^	10	5				1555 nm
quantum wells in InP-on-Si RRM^13^	4	1	0.01	10 aJ/bit	263	1598 nm
quantum wells in InP-on-Si RRM^13^	9	4	0.11	1.3 fJ/bit	263	1597 nm
SOH MZM on SOI^16^		140^a^	1.4	74 fJ/bit	1′000	1532 nm
SOH MZM on SOI^16^		10	0.23	2 fJ/bit	1′000	1532 nm
LiNbO_3_ on insulator MZM with superconducting electrodes^7^	17	4	0.01		200′000	1550 nm
this work: plasmonic organic MZM on SOI	>100	160^a^	0.90	5.6 fJ/bit	15	1528 nm
this work: plasmonic organic MZM on SOI	>100	16	0.10	230 aJ/bit	15	1528 nm
this work: plasmonic RRM on SOI	>67	256^a^	1.0	3.6 fJ/bit	123/10^c^	1285 nm

a The modulation format 4PAM was used instead of 2PAM.

b The following dimensions are stated for different modulator architectures: Circumference of the ring for RRMs; length of the active section per arm for MZMs.

c Length of the plasmonic phase shifter in the plasmonic RRMs.

## Conclusions

We demonstrate the viability of high-speed plasmonic OEO modulators operated at cryogenic temperatures. We show a reduction of the plasmonic propagation losses at 4 K by over 40% compared to room temperature, verifying for the first time reduced losses in a plasmonic waveguide at low temperatures. Next, we show a plasmonic MZM operated at cryogenic temperatures at 1528 nm wavelength. The device shows a setup-limited flat electro-optic bandwidth exceeding 100 GHz with no roll-off behavior at 4 K. Furthermore, we observe an increase of the half-wave voltage by only 11% compared to room temperature. Using this device, we demonstrate cryogenic electro-optic signal conversion by performing data transmission experiments with line rates up to 180 Gbit/s and energy-efficient transmission with an electrical energy consumption as low as 230 aJ/bit by using an electrical driving signal of 96 mV_pp_ at 16 Gbit/s. Finally, we demonstrate, for the first time, a plasmonic RRM in the O band at 1285 nm. The device modulates up to 256 Gbit/s using a 4PAM signal at cryogenic temperatures. This work shows that plasmonic OEO modulators not only feature high bandwidths at cryogenic temperatures but also are energy-efficient. In addition, they have lower plasmonic losses and enable the highest data rates. Thus, they are ideal building blocks for next-generation high-speed optical interconnects between room-temperature and cryogenic applications.

## Supporting Information Available

Device microscope image and measurement setup for plasmonic propagation loss, uncertainty estimation of plasmonic propagation loss, measurement data of plasmonic propagation loss, measurement setup for *V*_π_, measurement setup for electro-optic bandwidth, and active heat load of the plasmonic modulator (PDF)
