# Supplementary material for: Plasmonic Modulators in Cryogenic Environment Featuring Bandwidths in Excess of 100 GHz and Reduced Plasmonic Losses
Source: ACS Photonics. 2024 Jun 28;11(7):2691–9. doi: 10.1021/acsphotonics.4c00507 (PMC11258783; doi:10.1021/acsphotonics.4c00507)
Supplement: Supplementary file 1 — ph4c00507_si_001.pdf [file ph4c00507_si_001.pdf]

Supporting Information for

Plasmonic Modulators in Cryogenic Environment Featuring Bandwidths in Excess of 100 GHz and Reduced Plasmonic Losses

Dominik Bisang,[1], Yannik Horst[1], Maurus Thürig[1], Kiran Menachery[1], Stefan M. Koepfli[1], Manuel Kohli[1], Eva De Leo[2], Marcel Destraz[2], Valentino Tedaldi[2], Nino Del Medico[2], Claudia Hoessbacher[2], Benedikt Baeuerle[2], Wolfgang Heni[2], Juerg Leuthold*,[1,2]*

[1]ETH Zurich, Institute of Electromagnetic Fields, 8092 Zurich, Switzerland

[2]Polariton Technologies AG, 8134 Adliswil, Switzerland

*Corresponding Authors: dominik.bisang@ief.ee.ethz.ch, Juerg.Leuthold@ief.ee.ethz.ch

Supporting Note 1: Device Microscope Image and Measurement Setup for Plasmonic Propagation Loss

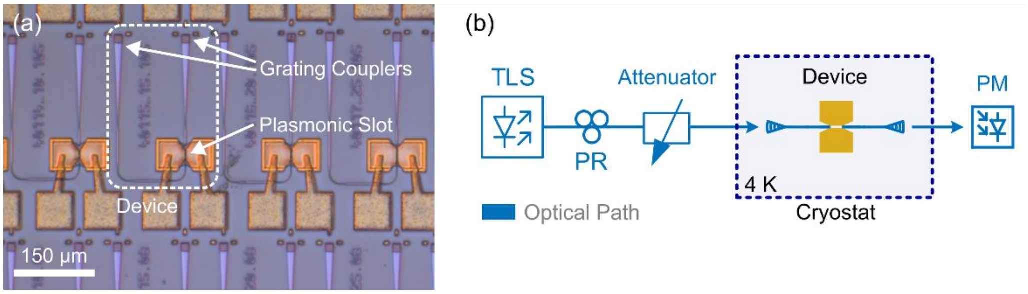

Figure S1. (a) Microscope image of an exemplary plasmonic slot device used for measuring the plasmonic losses. A single device consists of a plasmonic slot with specified slot width and slot length. Light is coupled from a fiber array into a waveguide by grating couplers, and from the waveguide into the plasmonic slot via a tapering section. The devices have RF electrical pads connected to use the devices as plasmonic phase shifters, however the pads were not used here. (b) Schematic drawing of the experimental setup to measure the plasmonic propagation loss in the cryostat. TLS, tunable laser source; PR, polarization rotator; DUT, device under test; PM, power meter.

Supporting Note 2: Uncertainty Estimation of Plasmonic Propagation Loss

The plasmonic propagation loss was fitted from pairs of data (x_i, y_i), where x_i is the length of the plasmonic slot and y_i is the measured fiber-to-fiber optical loss in decibel. A linear fit for the total optical loss $\hat{y}(x)$ as a function of the plasmonic slot length x was then performed according to:

$$\hat{y}(x) = \hat{a} \cdot x + \hat{b} \qquad \text{Eq. (1)}$$

where $\hat{a}$ corresponds to the estimated plasmonic propagation loss and $\hat{b}$ to the estimated optical insertion loss consisting of coupling loss from the fiber to the photonic waveguide, coupling loss from the photonic waveguide into the plasmonic slot, and (negligible) photonic propagation loss.

The *polyval* function of MATLAB was used for the fitting and getting an estimation of the covariance matrix of the fitting parameters $\hat{a}$ and $\hat{b}$. The variance $\sigma_{\hat{a}}^2$ of the estimated plasmonic propagation loss $\hat{a}$ needs however to be corrected for the fact that only a small number of data points n is available for the fit, usually $n = 5$.

For this, the t-value from the Student's t-distribution is calculated using a 1-sigma confidence level and n-2 degrees of freedom. For $n = 5$, this is approximately $t = 1.19$. The estimated uncertainty $\Delta\hat{a}$ of the plasmonic propagation loss is then given by:

$$\Delta\hat{a} = t \cdot \sigma_{\hat{a}} \qquad \text{Eq. (2)}$$

The plasmonic propagation loss in all figures is reported in the format $\hat{a} \pm \Delta\hat{a}$ in units dB/µm.

Supporting Note 3: Measurement Data of Plasmonic Propagation Loss

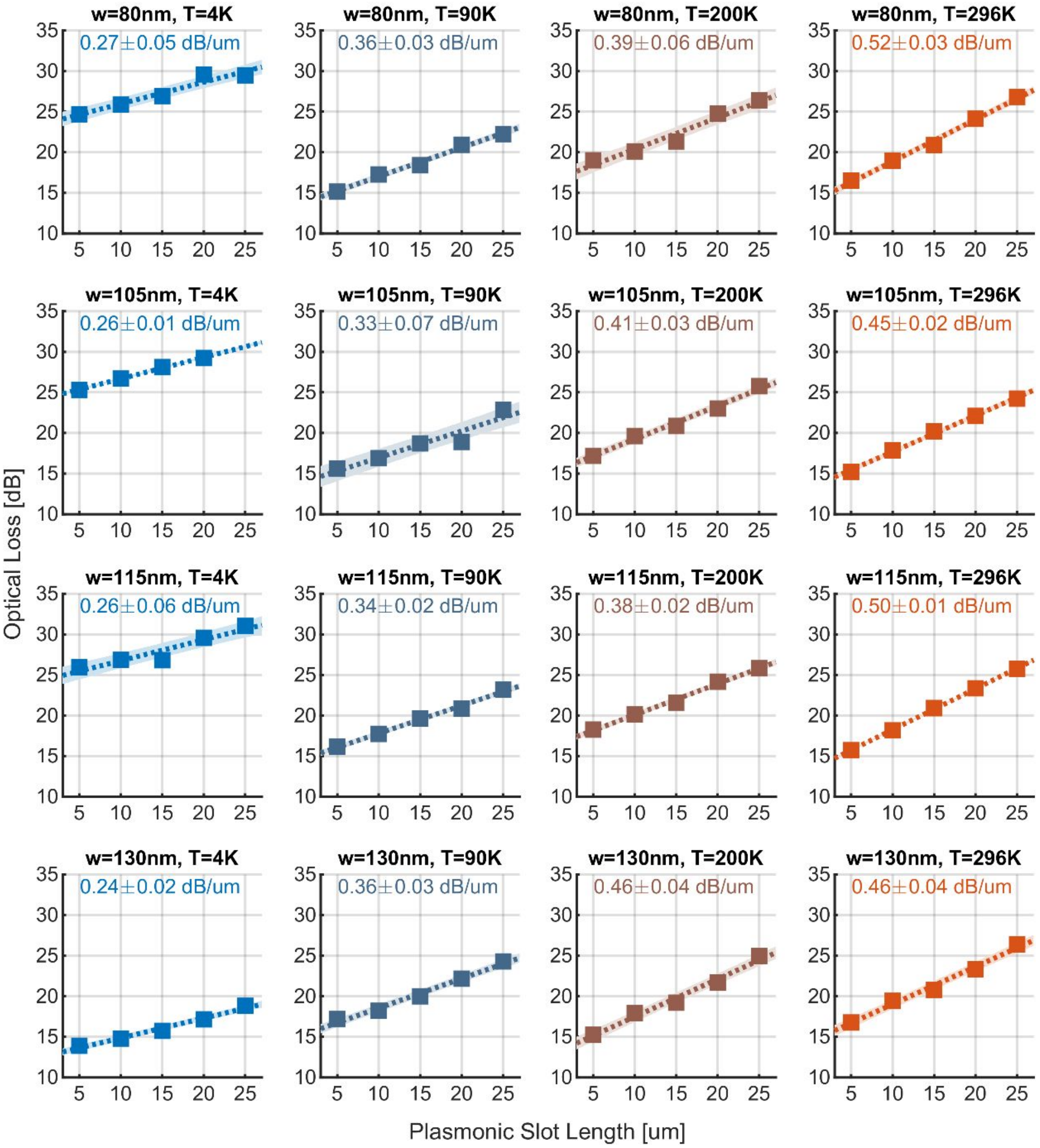

Figure S2. Measurement data to evaluate the plasmonic propagation loss at different temperatures and different plasmonic slot widths. Plots from left to right: In each row, the same devices are measured at 4 K, 90 K, 200 K and 296 K. Plots from top to bottom: Different devices with plasmonic slot widths of 80 nm, 105 nm, 115 nm, and 130 nm are measured. In each plot: The optical fiber-to-fiber insertion loss, including feedthrough into the cryostat and fiber-to-chip coupling, of five devices with different plasmonic slot length, but identical slot width, are shown. The dashed line is a linear fit through the measured data. The shadowed area surrounding the dashed line is the one-sigma confidence interval of the fit. The plasmonic propagation loss and the one-sigma uncertainty (text at the top of each plot) are extracted from the slope of the linear fit. The uncertainty estimation of the propagation loss is described in Supporting Note 2.

Supporting Note 4: Measurement Setup for Electro-Optic Bandwidth

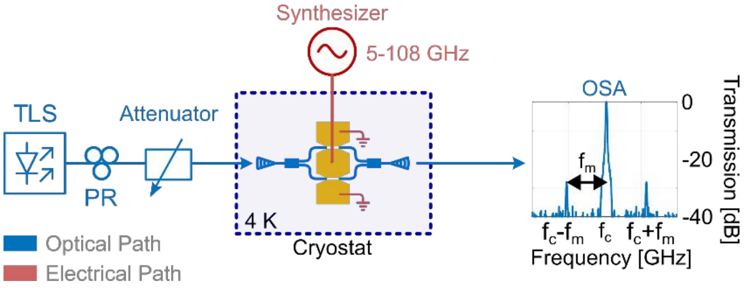

Figure S3. Schematic drawing of the experimental setup to measure the electro-optic bandwidth of the plasmonic MZM in the cryostat. The spectrum on the right is measured at $f_m = 70$ GHz small-signal RF modulation, the transmission is normalized to the carrier at frequency f_c. The optical carrier of the TLS was set to 1532.5 nm wavelength, operating the modulator in the quadrature point (3-dB point) for intensity modulation. The electrical sinusoidal signal was generated from a RF synthesizer up to 70 GHz, above it was created using a 6x frequency multiplier. The electrical signal was fed to the device through a 67 GHz vacuum RF feedthrough and a 67 GHz RF probe. The modulated optical signal was measured with an OSA, and subsequentially the peak-to-sideband ratio was extracted. The electrical loss calibration of the whole electrical path except the RF probe was performed at room temperature using an electrical signal analyzer (ESA) up to 108 GHz. However, the RF probe is only calibrated up to 67 GHz, above the RF probe loss was linearly extrapolated, thereby leading to higher oscillations, and potentially underestimating the RF losses. TLS, tunable laser source; PR, polarization rotator; OSA, optical spectrum analyzer.

Supporting Note 5: Measurement Setup for V_π

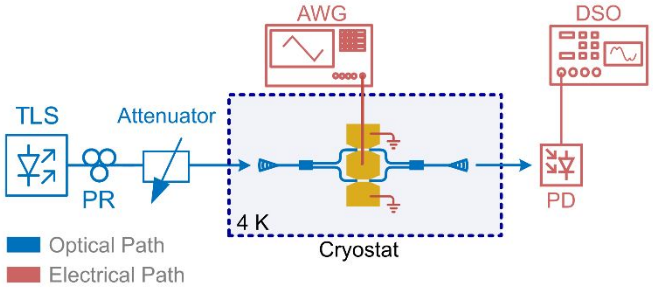

Figure S4. Schematic drawing of the experimental setup to measure the half-wave voltage V_π using the overmodulation method[1]. The AWG applies a 100 kHz triangular voltage with an amplitude exceeding the V_π of the modulator. The laser was operated between 1489 nm and 1567 nm with different operation wavelength at each temperature to compensate for a temperature-induced shift of the optimal operation point, which is due to a shift of the transmission spectrum at different temperatures. TLS, tunable laser source; PR, polarization rotator; AWG, arbitrary waveform generator; PD, photodiode; DSO, digital sampling oscilloscope.

Supporting Note 6: Active Heat Load of Plasmonic Modulator

Given the currently available cryostat technologies, the cooling power of a cryostat is typically in the order of 1 W at the 4 K stage[2]. Going to lower temperatures, the available cooling power is even further restricted. Therefore, it is crucial that any component inside a cryostat consumes as little power as possible.

Here, we briefly discuss the active heat load imposed by a plasmonic modulator for a cryogenic photonic link. Passive heat load from heat flux through any electrical cable or optical fiber connected to the modulator is omitted, since these are independent from the chosen modulator technology. The active heat load then consists of two parts, the electrical and optical power consumption. We will first discuss the electrical energy consumption, followed by the optical power consumption. There, we show that most optical power consumption arises from the fiber-to-chip coupling, and that improved coupling will lead to massively decreased power consumption for the plasmonic modulator. Finally, we show that it is possible to trade electrical and optical power consumption, while achieving the same BER in data-transmission.

The electrical power consumption is discussed in the main text, and strongly depends on the modulator design, electrical drive voltage, modulation format, and data transmission rate. For example, with the plasmonic RRM operated at 4 K and 1285 nm optical carrier wavelength, an electrical energy consumption for 16 GBd 2PAM as low as 475 aJ/bit was found for 191 mV drive voltage (see main text for the procedure to calculate electrical energy per bit). Having continuous data transmission without interruption, this corresponds to roughly $475\ \text{aJ/bit} \cdot 16\ \text{Gbit/s} \approx 7.6\ \mu\text{W}$ electrical power consumption.

Simultaneously, an optical carrier with 0 dBm power (1 mW) is launched into the cryostat, with -22 dB fiber-to-fiber loss measured for this device. For operation in the 3 dB working point, the corresponding optical power at the output is -25 dBm (3.1 µW). The remaining optical power of 0.997 mW ≈ 1 mW is lost inside the cryostat and contributes to the optical heat load. Therefore, the overall active heat load imposed by the plasmonic ring-resonator at 16 GBd 2PAM and 191 mV drive voltage is 7.6 µW + 0.997 mW ≈ 1 mW. Clearly, the active heat load of the measured devices is dominated by the optical loss inside the cryostat.

However, the active heat load can be reduced. The vast majority of optical losses are due to not yet optimized fiber-to-chip coupling losses. In our case, the fiber-to-fiber losses were -22 dB. A conceptually similar device showed on-chip losses of -1.2 dB at room temperature[3]. This is why we assume that the -22 dB fiber-to-fiber loss is mainly dominated by fiber-to-chip coupling. At room temperature, coupling efficiencies of <1 dB have already been demonstrated[4]. Assuming two times -1 dB fiber-to-chip coupling loss, -1.2 dB on-chip device losses, and a 3 dB working point loss, the optical loss would be improved from -25 dB to -6.2 dB, yielding 18.8 dB potential improvement. Then, the optical carrier power could be reduced by this amount while still achieving the same data-transmission performance, resulting in 13 µW optical power consumption. The overall heat load would then be 7.6 µW + 13 µW ≈ 21 µW.

For the plasmonic MZM, the optical power consumption is even higher than with the RRM since the measured insertion loss was -34 dB. Therefore, 10 dBm (10 mW) optical input power was used to compensate for these losses. However, having an estimated on-chip loss of -5.9 dB, the potential improvement in optical power consumption is even higher than with the RRM. If we again assume -1 dB per fiber-to-chip coupling, then the optical carrier power could in principle be reduced by 26.1 dB to achieve the same data-transmission performance.

In Table S1, we summarize the active heat load of the plasmonic MZM at 1528 nm and plasmonic RRM at 1285 nm for selected operation conditions as presented in this work. Further, for each case we calculate the active heat load for an ideal case where the fiber-to-chip coupling loss can be reduced to -1 dB per coupler. Assuming these coupling improvements and neglecting any passive heat load, then it is in principle possible to operate >30'000 plasmonic modulators at 16 GBd 2PAM with 1 W available cooling power, as it is typical for a 4 K stage.

Table S1. Active heat load of plasmonic MZM at 1528 nm and RRM at 1285 nm (4 K operation).

Device	Modulation Format and Baud Rate	Drive Voltage Peak-to-Peak	Electrical Power Consumption[a]	Optical Power Consumption (this work)	Active Heat Load (this work)	Optical Power Consumption (ideal)[b]	Active Heat Load (ideal)[b]
Plasmonic MZM	16 GBd 2PAM	96 mV	3.7 µW	10 mW	10 mW	24.5 µW	28.2 µW
Plasmonic MZM	128 GBd 2PAM	1000 mV	3.2 mW	10 mW	13.2 mW	24.5 µW	3.2 mW
Plasmonic MZM	80 GBd 4PAM	897 mV	894 µW	10 mW	10.9 mW	24.5 µW	919 µW
Plasmonic RRM	16 GBd 2PAM	191 mV	7.6 µW	1 mW	1 mW	13.1 µW	20.7 µW
Plasmonic RRM	128 GBd 2PAM	1000 mV	1.7 mW	2.7 mW	4.4 mW	35.4 µW	1.7 mW

Plasmonic RRM	180 GBd 2PAM	1000 mV	2.3 mW	2.7 mW	5.0 mW	35.4 µW	2.4 mW
Plasmonic RRM	128 GBd 4PAM	1000 mV	924 µW	2.7 mW	3.6 mW	35.4 µW	959 µW

[a]Electrical energy consumption per bit (see main text for procedure to calculate) multiplied with bit-rate.

[b]Assuming -1 dB fiber-to-chip coupling loss per coupler; and operation in 3 dB working point.

Finally, we want to note that for data-transmission, one can trade electrical drive voltage and optical input power to achieve the same signal-to-noise ratio (SNR), and thereby achieve similar bit-error-rate (BER). For example, in our measurements where the active heat load is mainly dominated by optical loss due to high fiber-to-chip coupling loss, it might be beneficial to reduce the optical input power but instead increase the electrical drive voltage.

This concept is illustrated in Figure S5, where 10 GBd 2PAM data-transmission experiments using the plasmonic RRM operated at 4 K and 1285 nm optical carrier wavelength are shown for different optical fiber input powers and electrical drive voltages $V_{PP,50\Omega}$. Figure S5a shows the measured SNR and Figure S5b the measured BER, both after digital signal-processing. Additionally, in Figure S5b, the HD-FEC limit (magenta line) and SD-FEC limit (red line) are indicated.

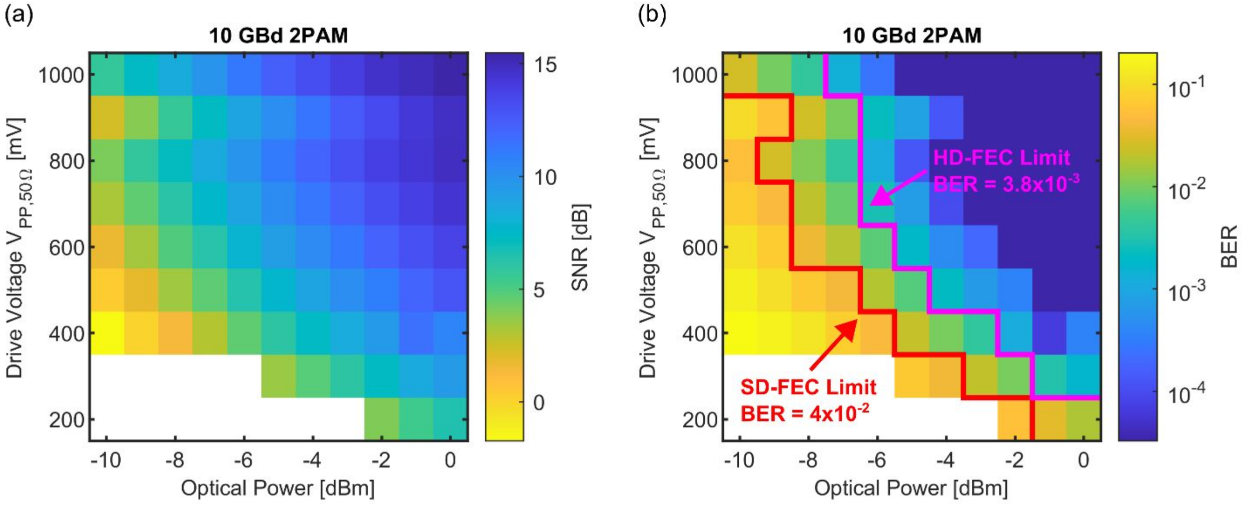

Figure S5. Data-transmission experiments using the plasmonic RRM operated at 4 K and 1285 nm optical carrier wavelength. (a) Measured SNR and (b) BER are shown for 10 GBd 2PAM transmission with different optical fiber input powers and electrical drive voltages $V_{PP,50\Omega}$. In (b), the HD-FEC limit (magenta line) and SD-FEC limit (red line) are shown. The white area in the bottom left of both plots was not measured.

Clearly, it is possible to reduce the optical input power and increasing the electrical drive voltage, while simultaneously achieve the same BER in data-transmission. Thereby, one might find the optimum operation condition and minimize the active heat load, allowing for either higher data rates or more devices within the limited cooling power.

Reference

(1) Heni, W.; Haffner, C.; Elder, D. L.; Tillack, A. F.; Fedoryshyn, Y.; Cottier, R.; Salamin, Y.; Hoessbacher, C.; Koch, U.; Cheng, B.; Robinson, B.; Dalton, L. R.; Leuthold, J. Nonlinearities of Organic Electro-Optic Materials in Nanoscale Slots and Implications for the Optimum Modulator Design. *Opt. Express* **2017**, *25* (3), 2627. https://doi.org/10.1364/oe.25.002627.

(2) Krinner, S.; Storz, S.; Kurpiers, P.; Magnard, P.; Heinsoo, J.; Keller, R.; Lütolf, J.; Eichler, C.; Wallraff, A. Engineering Cryogenic Setups for 100-Qubit Scale Superconducting Circuit Systems. *EPJ Quantum Technol.* **2019**, *6*. https://doi.org/10.1140/EPJQT/S40507-019-0072-0.

(3) Eppenberger, M.; Messner, A.; Bitachon, B. I.; Heni, W.; Blatter, T.; Habegger, P.; Destraz, M.; De Leo, E.; Meier, N.; Del Medico, N.; Hoessbacher, C.; Baeuerle, B.; Leuthold, J. Resonant Plasmonic Micro-Racetrack Modulators with High Bandwidth and High Temperature Tolerance. *Nat. Photonics 2023* **2023**, 1–8. https://doi.org/10.1038/s41566-023-01161-9.

(4) Vitali, V.; Domínguez Bucio, T.; Lacava, C.; Marchetti, R.; Mastronardi, L.; Rutirawut, T.; Churchill, G.; Faneca, J.; Gates, J. C.; Gardes, F.; Petropoulos, P. High-Efficiency Reflector-Less Dual-Level Silicon Photonic Grating Coupler. *Photonics Res.* **2023**, *11* (7), 1275. https://doi.org/10.1364/prj.488970.
